# Supplementary material for: Metabolomic Signature of Diabetic Kidney Disease in Cerebrospinal Fluid and Plasma of Patients with Type 2 Diabetes Using Liquid Chromatography-Mass Spectrometry
Source: Diagnostics (Basel). 2022 Oct 29;12(11):2626. doi: 10.3390/diagnostics12112626 (PMC9689120; doi:10.3390/diagnostics12112626)
Supplement: Supplementary file 1 [file diagnostics-12-02626-s001.zip › Figure S1.pdf]

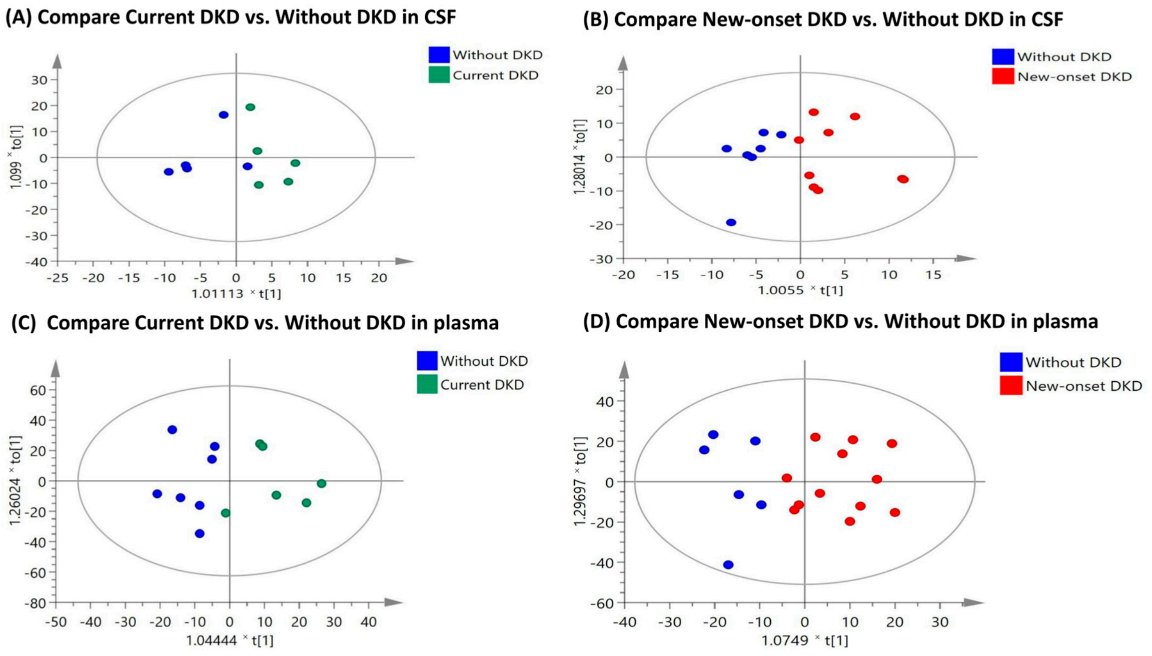

Figure S1: Orthogonal partial least-squares discriminant analysis (OPLS-DA) score plots in (A) CSF samples for comparison between patients with current DKD vs. without DKD (reliability: $R^2X = 0.611$, $R^2Y = 0.666$, $Q^2 = 0.139$), (B) new-onset DKD vs. without DKD (reliability: $R^2X = 0.599$, $R^2Y = 0.662$, $Q^2 = 0.034$). (C) plasma samples for comparison between patients with current DKD vs. without DKD (reliability: $R^2X = 0.496$, $R^2Y = 0.723$, $Q^2 = 0.246$, (D) patients with new-onset DKD vs. without DKD (reliability: $R^2X = 0.376$, $R^2Y = 0.718$, $Q^2 = 0.247$).
